# Proteomic Analysis of Adult *Ascaris suum* Fluid Compartments and Secretory Products

**DOI:** 10.1371/journal.pntd.0002939

**Published:** 2014-06-05

**Authors:** James F. Chehayeb, Alan P. Robertson, Richard J. Martin, Timothy G. Geary

**Affiliations:** 1 Institute of Parasitology and Centre for Host-Parasite Interactions, McGill University, Ste-Anne-de-Bellevue, Quebec, Canada; 2 Department of Biomedical Sciences, College of Veterinary Medicine, Iowa State University, Ames, Iowa, United States of America; University of Melbourne, Australia

## Abstract

**Background:**

Strategies employed by parasites to establish infections are poorly understood. The host-parasite interface is maintained through a molecular dialog that, among other roles, protects parasites from host immune responses. Parasite excretory/secretory products (ESP) play major roles in this process. Understanding the biology of protein secretion by parasites and their associated functional processes will enhance our understanding of the roles of ESP in host-parasite interactions.

**Methodology/Principal Findings:**

ESP was collected after culturing 10 adult female *Ascaris suum*. Perienteric fluid (PE) and uterine fluid (UF) were collected directly from adult females by dissection. Using SDS-PAGE coupled with LC-MS/MS, we identified 175, 308 and 274 proteins in ESP, PE and UF, respectively. Although many proteins were shared among the samples, the protein composition of ESP was distinct from PE and UF, whereas PE and UF were highly similar. The distribution of gene ontology (GO) terms for proteins in ESP, PE and UF supports this claim. Comparison of ESP composition in *A. suum*, *Brugia malayi* and *Heligmosoides polygyrus* showed that proteins found in UF were also secreted by males and by larval stages of other species, suggesting that multiple routes of secretion may be used for homologous proteins. ESP composition of nematodes is both phylogeny- and niche-dependent.

**Conclusions/Significance:**

Analysis of the protein composition of *A. suum* ESP and UF leads to the conclusion that the excretory-secretory apparatus and uterus are separate routes for protein release. Proteins detected in ESP have distinct patterns of biological functions compared to those in UF. PE is likely to serve as the source of the majority of proteins in UF. This analysis expands our knowledge of the biology of protein secretion from nematodes and will inform new studies on the function of secreted proteins in the orchestration of host-parasite interactions.

## Author Summary

*Ascaris lumbricoides*, the most prevalent metazoan parasite of humans, is a public health concern in resource-limited countries. Survival of this parasite in its host is mediated at least in part by parasite materials secreted into the host. Little is known about the composition of these secretions; defining their contents and functions will illuminate host-parasite interactions that lead to parasite establishment. *Ascaris suum*, a parasite of pigs, was used as a model organism because its genome has been sequenced and it is very closely related to *A. lumbricoides*. Excretory/secretory products (ESP), uterine fluid (UF) and perienteric fluid (PE) were collected from adult *A. suum*. Proteins were subjected to LC-MS/MS. ESP proteins (the ‘secretome’) included many also present in UF. Proteins in ESP but not in UF had considerably different characteristics than those in PE or UF, which were similar to each other. We conclude that proteins released from the secretory apparatus have distinct patterns of biological function and that UF proteins are likely derived from PE. Comparing the protein composition of *A. suum* ESP to ESP from *B. malayi* and *H. polygyrus* suggests that the secretome is conserved at the level of both phylogeny and host predilection site.

## Introduction

*Ascaris lumbricoides* is an extremely prevalent gastrointestinal nematode parasite of humans. Considered a neglected tropical disease (NTD) and widespread in populations of low-to middle-income countries, especially in tropical and subtropical regions, this parasite is estimated to infect as many as 1.2 billion people (many of whom harbor multiple species) in sub-Saharan Africa, China, South and Central America and East Asia [1]–[5]. Adult parasites reside primarily in the duodenum. Although infections are typically asymptomatic, detectable morbidity occurs in up to 200 million infections, most frequently in chronically infected individuals. Pathology may result when parasites migrate to the bile ducts, causing cholangitis. A high worm burden can obstruct the bowel and lead to volvulus and may cause pain, discomfort and megacolon. *Ascaris lumbricoides* has also been reported to cause lactose intolerance and to reduce absorption of vitamin A. Severe pathology can occur when larvae migrate through the lungs, causing inflammatory reactions. This may lead to pneumonitis, depending on the number of larvae penetrating the alveolar walls, and to pulmonary eosinophilia, with symptoms of fever and difficulty in breathing.

Chemotherapy remains the primary method of control for ascariasis, most commonly relying on albendazole or mebendazole [6]–[9]. Mass drug administration programs employing these drugs typically target school-age children once or twice a year. A single oral dose of albendazole, mebendazole or pyrantel pamoate is highly efficacious, but the distribution and incidence of this parasite remain very high.

Achieving a better understanding of how nematodes influence host immune responses to establish a chronic infection may lead to the development of novel control methods. The success of a parasite in establishing in a host depends on evading or modulating host immune responses, and understanding the molecular basis underlying this strategy is a compelling area of research [10]–[16]. These strategies are thought to be orchestrated through molecules released by parasites that shape the host-parasite interaction. Recently, the protein composition of nematode excretory/secretory products (ESP) has been characterized in several species using proteomics approaches based on mass spectrometry. Species studied include *Brugia malayi* [14], [17], [18], *Heligmosoides polygyrus* [19], [20], *Ancylostoma caninum* [21], *Meloidogyne incognita* [22], [23], *Strongyloides ratti* [24] and *Dirofilaria immitis* [25].

Despite its importance as a highly prevalent NTD, limited knowledge is available about the biology of ESP in *A. lumbricoides*. Because *A. suum* is very closely related to *A. lumbricoides* and its genome has been published [26], we characterized the protein composition of perienteric fluid (PE), uterine fluid (UF) and total ESP (the secretome) from this parasite. The large size of this species makes it possible to gain insights into the origin of proteins in ESP and the relative contribution of proteins released from the uterus during egg shedding.

## Materials and Methods

### Parasite culture and fluid collection

Adult *A. suum* were obtained from JBS Swift and Co. pork processing plant, Marshalltown, Iowa, USA. They were maintained in Locke's solution (NaCl 155 mM, KCl 5 mM, CaCl_2_ 2 mM NaHCO_3_ 1.5 mM, glucose 5 mM) at 32°C. ESP collections were carried out within 24 hr of procurement by maintaining 10 large active females in 500 ml fresh Locke's solution. PE fluid was collected by snipping the head off adult nematodes with a fine scissors so that the turgor pressure discharged the fluid directly into a 1.5 ml screw-capped tube. Approximately, 300 µl of UF was collected by positioning the ovijector over a similar tube and then gently compressing each end of the parasite toward the opening to expel fluid and eggs. The thick nature of UF required 1∶1 dilution with sterile phosphate-buffered saline, pH 7.6, for processing after collection. All solutions were stored in sterile 1.5 ml microtubes and shipped overnight on dry ice to the Institute of Parasitology, McGill University, for further analysis.

### Sample preparation

ESP, PE and UF fluids were collected from 10 nematodes and pooled. A mixture of protease inhibitors [bestatin hydrochloride; 4-(2-aminoethyl) benzenesulfonyl fluoride hydrochloride; N- (trans-epoxysuccinyl)-L- leucine 4-guanidinobutylamide; phosphoramidon disodium salt; pepstatin A; Sigma, St. Louis MO) was added to PE and ESP samples. The ESP sample was sterilized by passage through a 0.22 µm filter and concentrated with an Amicon (MWCO 3000) ultrafiltration as described elsewhere [17]. Only proteins in the ESP sample were precipitated with cold trichloroacetic acid (TCA; final concentration 20%). TCA pellets were washed with cold acetone (−30C) and air-dried. Pellets were suspended in Tris-HCl (20mM, pH 8.0) and concentration measured (Quant-iTTM Protein Assay, Invitrogen). UF and PE were centrifuged at 8, 000×g for 10 min, which pelleted eggs from UF, and the supernatants collected for analysis. All samples were stored at −80C until further analysis.

### Gel electrophoresis

Protein pellets were dissolved in Laemmli buffer (50 mM Tris-HCl, 2% SDS, 10% glycerol, 1% β-mercaptoethanol, 12.5 mM EDTA, 0.02 % bromophenol blue, pH 6.8). Aliquots of 40 µl were loaded on a precast gradient gel (4–12%, 10×10 cm; Invitrogen). The gel was stained with AgNO_3_ using standard protocols. ESP, PE and UF lanes were cut into 1 mm×1 cm slices with a razor blade under a laminar flow hood, which were incubated in 30 mM potassium ferricyanide, 100 mM sodium thiosulfate (1∶1) for 5 min. After 3 washes with water, the bands were incubated in ACN for 10 min and prepared for trypsin digestion and mass spectrometry.

### Mass spectrometry

The gel slices were destained with 50% methanol, reduced in 10 mM DTT for 1 hr at 56 C and alkylated in 55 mM chloroacetamide for 1 hr at room temperature. After washing in 50 mM ammonium bicarbonate, gel pieces were shrunk in 100% ACN. Trypsin (Promega) digestion (100 ng in 50 mM ammonium bicarbonate) was conducted for 8 hr at 37 C. Peptides were extracted in 90% ACN/0.5 M urea and dried in a speed vac. Samples were resolubilized in 5% ACN/ 0.2% formic acid and separated on a laboratory-made C_18_ column (150 µm×10 cm) using an Eksigent nanoLC-2D system. A 56-min gradient from 5–60% ACN (0.2% FA) was used to elute peptides from a homemade reversed-phase column (150 µm×100 mm) with flow rate  = 600 nl/min. The column was directly connected to a nanoprobe interfaced with an LTQ-Orbitrap Elite mass spectrometer (Thermo-Fisher). Each full MS spectrum was followed by 12 MS/MS spectra (13 scan events) from which the 12 most abundant multiply charged ions were selected for MS/MS sequencing. Tandem MS experiments were performed using collision-induced dissociation in the linear ion trap. The data were processed using the 2.3 Mascot (Matrix Science) search engine with tolerance parameters set to 15 ppm and 0.5 Da for the precursor and the fragment ions respectively. The selected variable modifications were carbamidomethyl (C), deamidation (NQ), oxidation (M) and phosphorylation (STY). The database was the *Ascaris suum* genome draft located at [ftp://ftp.wormbase.org/pub/wormbase/species/a_suum/assemblies/v1/Ascaris_suum_geneset_annotated.pep].

### Data analysis

Tandem mass spectra were extracted, charge state deconvoluted and deisotoped. All MS/MS samples were analyzed using Mascot (Matrix Science, London, UK; version 2.3.01). Mascot was set up to search the gs_asc201204 database (selected for All Entries, 201204, 18542 entries) assuming the digestion enzyme, trypsin. Mascot was searched with a fragment ion mass tolerance of 0.50 Da and a parent ion tolerance of 7.0 ppm. The iodoacetamide derivative of cysteine was specified in Mascot as a fixed modification. Oxidation of methionine was specified in Mascot as a variable modification. A criterion for protein identification - Scaffold (Version Scaffold_4.2.0, Proteome Software Inc., Portland, OR) was used to validate MS/MS based peptide and protein identifications. Peptide identifications were accepted if they could be established at greater than 95.0% probability as specified by the Peptide Prophet and contained at least 2 identified peptides algorithm [27]. Protein probabilities were assigned by the Protein Prophet Algorithm [28], which within the Scaffold proteome platform predicted a false discovery rate (FDR) of 0 %. Proteins that contained similar peptides and could not be differentiated based on MS/MS analysis alone were grouped to satisfy the principles of parsimony.

### Protein characterization: Bioinformatics

Blast2GO software was used to acquire gene annotations [29]. Using default parameters, a BLASTP search was performed versus the entire non-redundant NCBI amino acid database using 1×10^−3^ as the minimum expectation value and a 33 cut-off for high-scoring segment pairs. Default parameters were used for annotations while setting the value of pre-eValue-Hit-Filter to 1×10^−6^, a cut-off value of 55 for annotation and 5 for gene ontology (GO) weight. Annotation Expander (ANNEX), integrated within Blast2GO, was essential to augment annotations [30]. InterPro database scans were performed within Blast2GO to retrieve functional domains and GO terms, both essential to complement annotation. Retrieved GO terms and InterPro results were merged to add domains and motifs to annotations [31]. GO terms were filtered (in percentage) in Blast2GO by the number of sequences collected for each. ESP and UF biological processes (level 2) GO terms were each filtered at 2%. ESP, ESP-UF, PE and UF biological processes (level 4) were filtered at 8%, 5%, 35% and 40%, respectively. UF molecular functions (level 2) were filtered at 2%. ESP, ESP-UF, PE and UF molecular functions (level 4) were filtered at 5%, 5%, 12% and 8%, respectively. Proteins in ESP, PE and UF were scanned for N-terminal signal peptides using SignalP 4.1 [32].

## Results

### Protein identification

**Figure 1:**
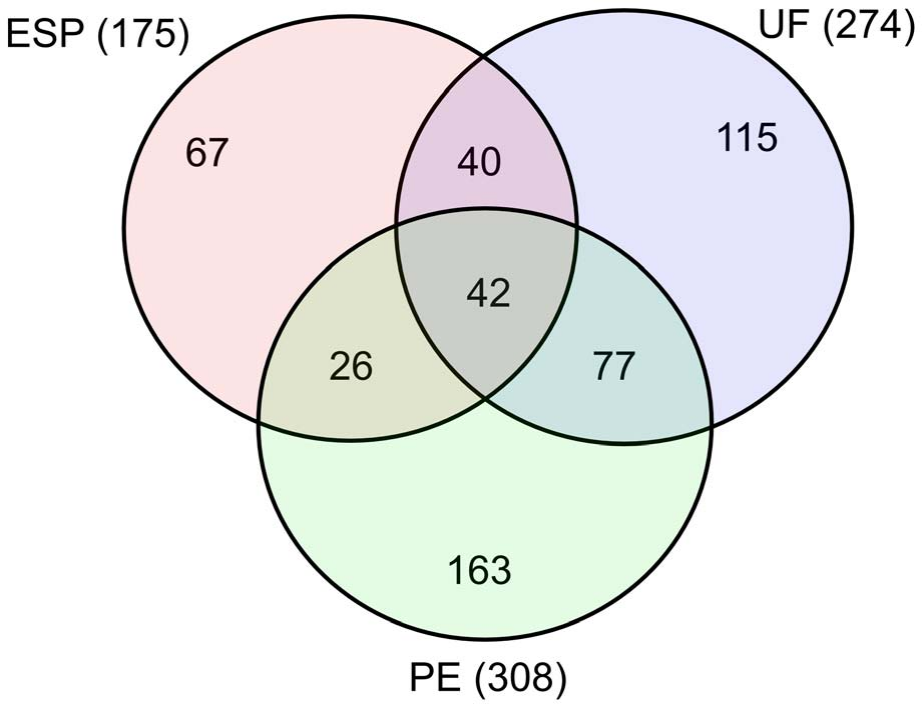
Distribution of proteins among ESP, PE and UF. The Venn diagram shows the numbers of proteins shared between and among the three compartments based on common Accession Number.

We obtained ∼45 µg protein in the ESP sample, ∼60 µg in UF and ∼55 µg in PE, all of which was used for protein content analysis. We identified 530 distinct proteins in these samples, 175 in ESP, 308 in PE and 274 in UF. The 20 most abundant proteins in these samples are shown in Tables 1–4, with the complete lists provided in Tables S1–S3. In the ESP sample, 67 proteins were specific to ESP only, 26 were shared only between ESP and PE, and 40 were common only to ESP and UF (Table S4). Forty-two proteins (8%) were common among the three compartments; 163 (31%) were appointed solely to PE, 115 (22%) were specific to UF and 77 (15%) were shared between PE and UF (Figure 1).

**Table 1:** The twenty most abundant proteins in ESP from *Ascaris suum*.

#	Accession	Protein Name	SP	NCBI Accession	BlastP result	Organism
1	GS_20234	Polyprotein ABA-1	Y	AAD13651.1	ABA-1 allergen	*A. lumbricoides*
2	GS_08371	Glb-1	Y	P28316.2	Extracellular globin	*A. suum*
3	GS_08981	Protein Y75B7AR.1	Y	ERG84500.1	Hypothetical protein ASU_05287	*A. suum*
4	GS_11978	OV17	Y	ERG85519.1	Hypothetical protein ASU_03636	*A. suum*
5	GS_19115	Serpin B	N	ERG78895.1	Putative serpin-like protein ma 3388	*A. suum*
6	GS_02689	Serpin B	N	ERG78895.1	Putative serpin-like protein ma 3388	*A. suum*
7	GS_06453	Brugia malayi antigen	N	BAC66614.1	As16	*A. suum*
8	GS_05746	Peptidase - Family M1 unassigned peptidases	Y	BAC83078.1	Aminopeptidase N	*A. suum*
9	GS_05201	Major sperm protein 2	N	P27439.3	Major sperm protein isoform alpha	*A. suum*
10	GS_13011	Hypothetical protein	N	-	-	*-*
11	GS_07489	BM20	Y	ERG87764.1	Fatty-acid and retinol-binding protein 1	*A. suum*
12	GS_19373	Vitllogenin-4 (Vit-4)	Y	ERG79133.1	Vitellogenin-6	*A. suum*
13	GS_11183	MFP2	N	AAP94886.1	MFP2	*A. suum*
14	GS_08343	Collagen alpha-5	Y	ERG78661.1	C-type lectin protein	*A. suum*
15	GS_12601	Brugia malayi antigen	Y	ACJ03764.1	Ag1	*A. lumbricoides*
16	GS_07456	Transthyretin-8 (Ttr-8)	Y	ERG85900.1	Transthyretin-like protein 5	*A. suum*
17	GS_19777	Probable alpha/beta-glucosidase agdC	N	ERG80708.1	Sucrase-intestinal	*A. suum*
18	GS_15314	IgGFc-binding protein	N	ERG867007.1	Apolipophorin	*A. suum*
19	GS_11674	Apolipophorins	N	ERG86007.1	Apolipophorin	*A. suum*
20	GS_16477	Hypothetical protein	N	-	-	*-*

“-“ indicates that a BLASTP search returned no significant match. “SP” refers to signal peptide; “Y” and “N” indicate the presence or absence of a signal peptide, respectively.

**Table 2:** The twenty most abundant proteins in UF from *Ascaris suum*.

#	Accession	Protein Name	SP	NCBI Accession	BlastP result	Organism
1	GS_20234	Polyprotein ABA-1	Y	AAD13651.1	ABA-1 allergen	*A. lumbricoides*
2	GS_15314	IgGFc-binding protein	N	ERG867007.1	Apolipophorin	*A. suum*
3	GS_19373	Vitllogenin-4 (Vit-4)	Y	ERG79133.1	Vitellogenin-6	*A. suum*
4	GS_01900	Methylmalonyl-CoA decarboxylase	N	ERG82671.1	Propionyl-carboxylase alpha	*A. suum*
5	GS_15475	Phosphoenolpyruvate carboxykinase	N	ERG79462.1	Phosphoenolpyruvate carboxykinase	*A. suum*
6	GS_14231	Glutamate dehydrogenase	N	ERG83952.1	Glutamate dehydrogenase	*A. suum*
7	GS_06246	Phosphoglycerate kinase	N	ERG83027.1	Putative phosphoglycerate kinase	*A. suum*
8	GS_21097	Fumarate hydratase	N	ERG84110.1	Putative fumarate hydratase	*A. suum*
9	GS_21295	Enolase	N	ERG79934.1	Enolase	*A. suum*
10	GS_11183	MFP2	N	AAP94886.1	MFP2	*A. suum*
11	GS_08789	Probable citrate synthase	N	ERG83688.1	Putative citrate synthase	*A. suum*
12	GS_14262	P40	N	AAP94889.1	MFP2b	*A. suum*
13	GS_10682	P40	N	AWW29197.1	MFP2c	*A. suum*
14	GS_11674	Apolipophorin	N	ERG86007.1	Apolipophorin	*A. suum*
15	GS_09975	Fructose-bisphosphate aldolase 1	N	ERG82018.1	Fructose-bisphosphate aldolase 1	*A. suum*
16	GS_14525	Methylmalonyl-CoA mutase	N	ERG80125.1	Putative Methylmalonyl-CoA mutase	*A. suum*
17	GS_03297	Protein disulfide-isomerase a3	Y	ERG84937.1	Protein disulfide-isomerase a3	*A. suum*
18	GS_09492	L-threonine 3-dehydrogenase	N	ERG80509.1	Sorbitol dehydrogenase	*A. suum*
19	GS_24173	Peptidase - subfamily S1A unassigned peptidases	N	ERG83341.1	Trypsin family protein	*A. suum*
20	GS_23848	Glutamate dehydrogenase	N	ERG82853.1	Glutamate dehydrogenase	*A. suum*

“SP” refers to signal peptide; “Y” and “N” indicate the presence or absence of a signal peptide, respectively.

**Table 3:** The twenty most abundant proteins in PE from *Ascaris suum*.

#	Accession	Identified Proteins (308)	SP	NCBI Accession	BlastP result	Organism
1	GS_20234	Polyprotein ABA-1	Y	AAD13651.1	ABA-1 allergen	*A. suum*
2	GS_11674	Apolipophorin	N	ERG86007.1	Apolipophorin	*A. suum*
3	GS_10956	Vitellogenin-5	N	ERG83573.1	Vitellogenin-6	A. suum
4	GS_15314	IgGFc-binding protein	Y	ERG867007.1	Apolipophorin	*A. suum*
5	GS_19373	Vit-4 protein	N	ERG79133.1	Vitellogenin-6	*A. suum*
6	GS_04737	Vitellogenin	N	ERG86007.1	Apolipophorin	*A. suum*
7	GS_03797	Heat shock 70 kDa protein 1	Y	ADI54942.1	Heat shock protein 70	*D. medinensis*
8	GS_17449	Molecular chaperone HtpG	N	ACO55134.1	Heat shock protein-90	*A. suum*
9	GS_21295	Enolase	N	ERG79934.1	Enolase	*A. suum*
10	GS_08371	GLB-1 protein	N	P28316.2	Hemoglobin	*A. suum*
11	GS_06246	Phosphoglycerate kinase	Y	ERG83027.1	Putative phosphoglycerate kinase	*A. suum*
12	GS_05301	Phosphoenolpyruvate carboxykinase [GTP]	N	ERG87334.1	Phosphoenolpyruvate carboxykinase	*A. suum*
13	GS_19115	Serpin	Y	ERG78895.1	Putative serpin-like protein ma 3388	*A. suum*
14	GS_19276	Fructose-bisphosphate aldolase	Y	ERG79663.1	Fructose-bisphosphate aldolase 1	*A. suum*
15	GS_11347	Starch phosphorylase	Y	ERG79023.1	Glycogen muscle form	*A. suum*
16	GS_18098	Aldo/keto reductase family protein	N	XP_003143212.1	Oxidoreductase	*Loa loa*
17	GS_23993	Tubulin beta	N	AGM37949.1	Beta-tubulin	*P.s equorum*
18	GS_17648	Phosphomannomutase	N	EJW88303.1	Phosphoglucomutase/phosphomannomutase domain-containing protein	*W. bancrofti*
19	GS_07489	BM20	N	ERG87764.1	Fatty-acid and retinol-binding protein 1	*A. suum*
20	GS_05590	Tyrosine 3-monooxygenase/tryptophan 5-monooxygenase activation protein	N	AHJ11135.1	14-3-3 zeta	*A. suum*

“SP” refers to signal peptide; “Y” and “N” indicate the presence or absence of a signal peptide, respectively.

**Table 4:** The twenty most abundant proteins in ESP-UF from *Ascaris suum*.

#	Accession	Protein Name	SP	NCBI Accession	BlastP result	Organism
1	GS_06453	*Brugia malayi* antigen	N	BAC66614.1	As16	*A. suum*
2	GS_05746	Peptidase - Family M1 unassigned peptidases	Y	BAC83078.1	Aminopeptidase N	*A. suum*
3	GS_12601	*Brugia malayi* antigen	Y	ACJ03764.1	Ag1	*A. lumbricoides*
4	GS_07456	Transthyretin-8 (Ttr-8)	Y	ERG85900.1	Transthyretin-like protein 5	*A. suum*
5	GS_17977	Probable alpha/beta-glucosidase agdC	N	ERG85169.1	T family of potassium channels protein 12	*A. suum*
6	GS_16078	Protein Y75B7AR.1	N	-	-	*-*
**7**	GS_07759	Protein C28C12.4	Y	ERG83483.1	Hypothetical protein ASU_06904	*A. suum*
8	GS_23855	Zinc Finger protein 420	Y	ERG80621.1	Hypothetical protein ASU_11569	*A. suum*
9	GS_08951	Hypothetical protein	N	ERG86760.1	Hypothetical protein ASU_01744	*A. suum*
10	GS_21887	Protein F55H12.4	Y	XP_003112257.1	Hypothetical protein CRE_29767	*C. remanei*
11	GS_17143	Protein T18B10.2	Y	ERG79357.1	Hypothetical protein ASU_13792	*A. suum*
12	GS_14400	Hypothetical protein	Y	-	-	*-*
13	GS_02066	Fab1, identical	Y	P55776.1	Fatty acid-binding protein homolog	*A. suum*
14	GS_01881	Transthyretin-8 (Ttr-8)	Y	ERG85900.1	Transthyretin-like protein	*A. suum*
15	L3E_02632	Transthyretin-8 (Ttr-8)	Y	ERG85638.1	Transthyretin-like protein	*A. suum*
16	GS_18848	Glutamate dehydrogenase	N	ERG83952.1	Glutamate dehydrogenase	*A. suum*
17	GS_20676	Annexin	N	ERG80393.1	Annexin A6	*A. suum*
18	GS_05584	Aminopeptidase N	N	ERG83077.1	Aminopeptidase N	*A. suum*
19	GS_02555	Aminopeptidase N	N	ERG83079.1	Aminopeptidase N	*A. suum*
20	GS_04373	L-isoaspartate O-methyltransferase	N	XP_001902004.1	L-isoaspartate O-methyltransferase	*B. malayi*

“-“ indicates that a BLASTP search returned no significant match. “SP” refers to signal peptide; “Y” and “N” indicate the presence or absence of a signal peptide, respectively.

### Protein abundance

Estimates of protein abundance are based on the number of peptides and assigned spectra and peptides from Scaffold [33]. For comparison, we subtracted proteins found in UF from those detected in ESP (ESP-UF) to segregate proteins presumably released through the ES system from those that originate from egg shedding. In support of this assumption, UF proteins detected in ESP were generally among the most abundant in UF (Table S3). Screening the ESP tryptic peptide data against all GenBank proteins identified hits against mammalian keratin and a few bacterial proteins in low abundance in ESP, PE and UF (data not shown), which were automatically excluded from the results.

Polyprotein ABA-1 (UniProt ID: F1KUF9) was among the most abundant proteins in all samples (Table 1–3). A protein related to the *Onchocerca volvulus* OV-17 antigen was abundant in both ESP and UF, suggesting that it may be derived from UF, whereas several homologs of *B. malayi* antigens were only present in ESP-UF. The high relative abundance of these antigens suggests that proteins in adult female ESP are derived from both the release of UF during egg shedding and the ES apparatus, a finding supported by the appearance of OV-17 and major sperm protein 2 in the ESP fraction. Significant overlap in the list of most abundant proteins between UF and PE suggests that the former is derived from the latter compartment and that both can be distinguished from ESP *per se* (ESP-UF). The ESP-UF subset was characterized by high abundance of hypothetical proteins compared to PE and UF. In contrast, glycolytic enzymes were much more abundant in PE and UF than in ESP-UF.

### Gene ontology

Blast2GO was used to extract GO terms for 134 proteins in ESP, 291 in PE, 264 in UF and 65 in ESP-UF.

**Figure 2:**
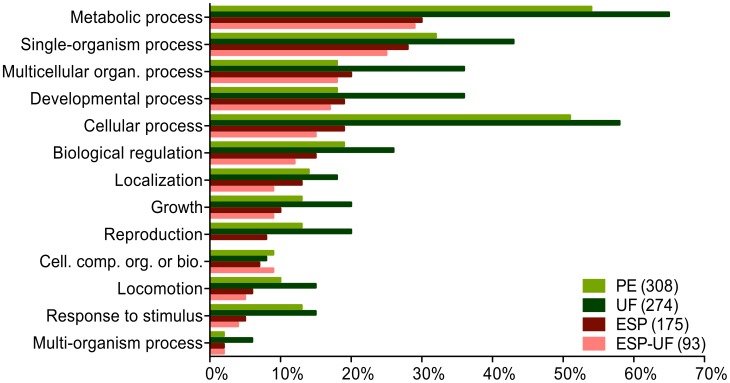
Distribution of level 2 molecular functions GO terms for proteins from UF, PE, ESP and ESP-UF. The three fractions are represented by different colors. ESP-UF refers to the proteins found in ESP but not in UF.

**Figure 3:**
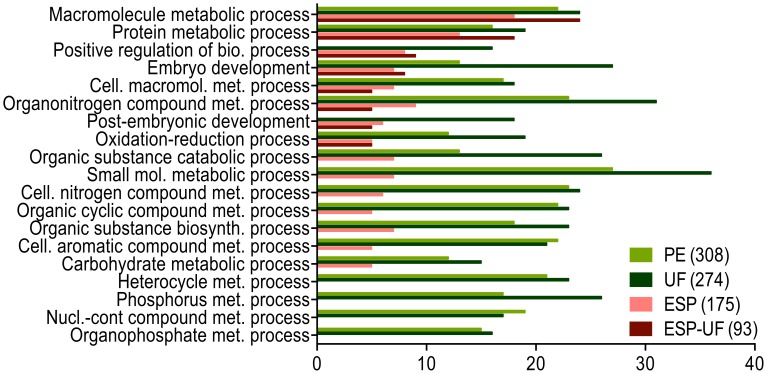
Distribution of level 4 molecular functions GO terms for proteins from UF, PE, ESP and ESP-UF. The three fractions are represented by different colors. ESP-UF refers to the proteins found in ESP but not in UF.

The molecular functions “binding” and “catalytic activity” were commonly predicted for proteins in UF, PE, ESP and ESP-UF (Figure 2). Higher-level molecular functions GO terms revealed subcategories of binding activity, such as “anion binding”, “nucleotide binding”, “nucleoside binding” and “nucleoside phosphate binding”, which dominated in PE and UF compared to ESP and ESP-UF (Figure 3). Although “peptidase activity” and “cation binding” were shared between the three sets of proteins, they were much more common in ESP-UF. Interestingly, “peptidase inhibitor activity” and “endopeptidase regulatory activity” were only found in ESP and ESP-UF.

**Figure 4:**
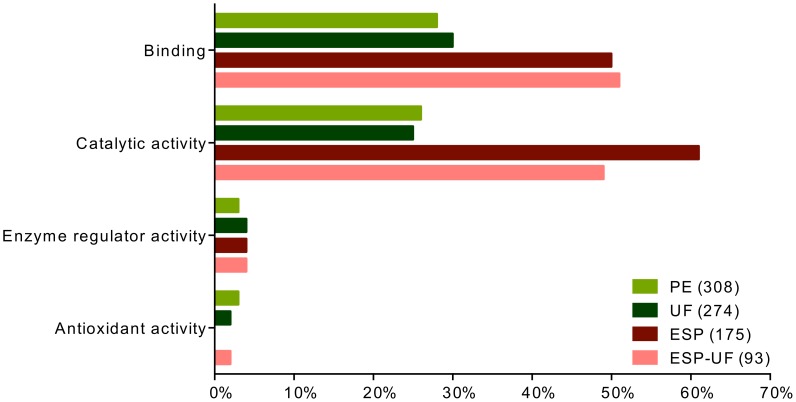
Distribution of level 2 biological processes GO terms for proteins from UF, PE, ESP and ESP-UF. The three fractions are represented by different colors. ESP-UF refers to the proteins found in ESP but not in UF.

**Figure 5:**
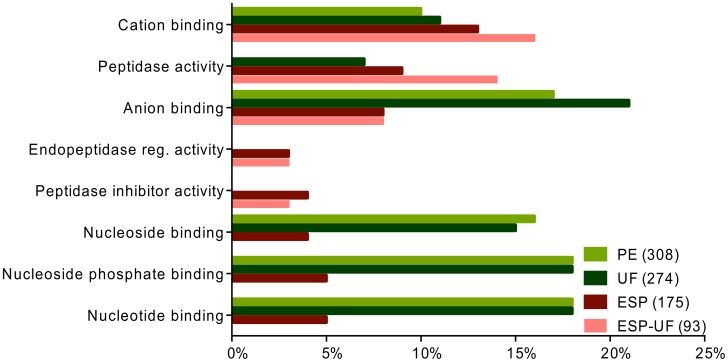
Distribution of level 4 biological processes GO terms for proteins from UF, PE, ESP and ESP-UF. The three fractions are represented by different colors. ESP-UF refers to the proteins found in ESP but not in UF.

Major biological function categories were “metabolic process”, “cellular process” and “single-organism process” in PE and UF (Figure 4). ESP and ESP-UF were similar in almost all GO categories. On a higher level of biological function analysis, most GO terms fell under cellular and metabolic processes (Figure 5), especially those from PE and UF. Cellular processes were allocated to “small molecule metabolic process”, “organic substance biosynthetic process” and similar GO terms with cellular processes activity (Figure 5).

### Signal peptide

All proteins in ESP, PE and UF were scanned for N-terminal signal peptide sequence using Signal P 4.1 [32]. Seventy protein sequences (40%), 57 (19%) and 39 (14%) had a signal peptide in ESP, PE and UF, respectively (Tables S1–S3).

### Comparative analysis

We previously analyzed ESP obtained in similar protocols from cultures of the Clade III nematode *B. malayi* [18] and the Clade V species *H. polygyrus*, which resides in the same general intestinal niche as *A. suum* [20]. We considered two samples to contain the same protein based on the name assigned to it in the Scaffold annotation, as confirmed by BlastP. The presence of >1 sequence with the same annotation in a sample was not a factor in the analysis. Because *A. suum* larval ESP was not investigated, we restricted the analysis to ESP from adult *B. malayi*.

**Figure 6:**
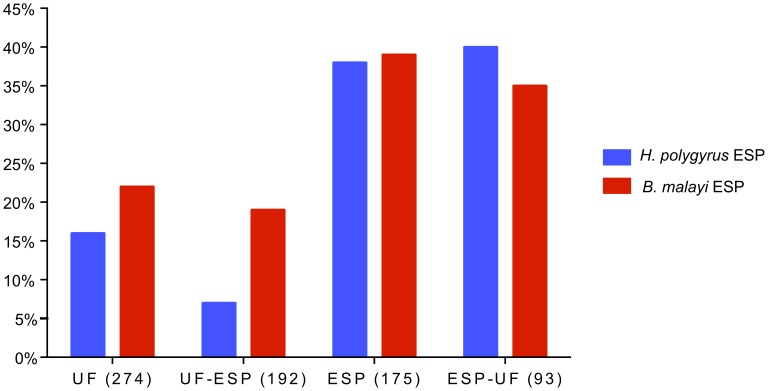
Percentage of protein similarity among UF, UF-ESP, ESP and ESP-UF from female *A. suum* and ESP from adult B. *malayi.* Percent similarity among UF, UF-ESP, ESP and ESP-UF from female *A. suum* and female, male and total adult *B. malayi* ESP. The comparison is based on annotation names for proteins in the various compartments, confirmed by BlastP analysis.

Comparison of *A. suum* UF and ESP to *B. malayi* ESP from female and male (separately and together) based on protein composition revealed that female *B. malayi* ESP was more similar to UF (UF-ESP: 14%) than to ESP (ESP-UF: 7%) from *A. suum* (Figure 6). Interestingly, protein composition of ESP from male *B. malayi* equally related to that of UF (UF-ESP: 18%) and ESP (ESP-UF: 18%) from *A. suum*. Overall, *B. malayi* adult ESP was more similar to *A. suum* UF (UF-ESP: 16%) than ESP (ESP-UF: 6%).

**Figure 7:**
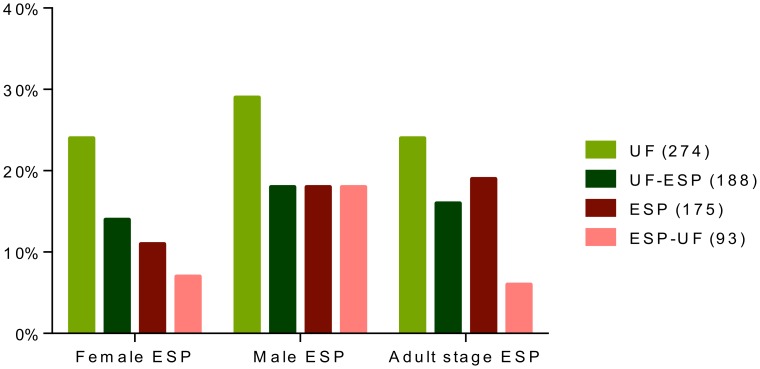
Cross-species comparisons of secreted proteins. Depicted is percent similarity of proteins in UF, UF-ESP, ESP and ESP-UF from *A. suum* with ESP from *B. malayi* and *H. polygyrus*. Comparisons are based on annotation names for proteins in the various samples, confirmed by BlastP analysis.

The same approach was used to compare ESP from *A. suum*, *B. malayi* and *H. polygyrus* (Figure 7). UF (UF-ESP) from *A. suum* was more similar to ESP from *B. malayi* (19%) than to ESP from *H. polygyrus* (7%) (Figure 7). In contrast, ESP-UF from *A. suum* was more similar to ESP from *H. polygyrus* (25%) than to ESP from *B. malayi* (13%) (Figure 7).

## Discussion

Establishment or resolution of a nematode infection is determined by molecular negotiations at the host-parasite interface, and it is a long-standing goal of parasitologists to identify the parasite and host molecules which determine the outcome of infection. It is generally accepted that parasite proteins make up at least part of the tactical strategy used to overcome host immune responses. Parasite secreted proteins also play essential roles in migration through the host, establishment in a preferred location for reproduction, and acquisition of nutrients. Nonetheless, we understand very little about the roles of specific parasite-derived proteins in the infection process. The very large number of proteins detected in nematode secretomes precludes a systematic evaluation of their biological roles, necessitating additional analyses to prioritize them for intensive experimental investigation. In this regard, *A. suum* is a useful experimental model, as its large size permits the selective determination of proteins in ESP based on their anatomical origin. The very close phylogenetic relationship of this species to the human-selective species *A. lumbricoides*, the most widespread helminth parasite of humans, further justifies this analysis.

Parasite proteins released into the host, excluding those derived from damaged or dying specimens, may be derived from classical excretory/secretory structures, from cuticle turnover, from the products of digestion in the parasite intestinal tract, and from the release of fluids during egg shedding and insemination. As immunomodulatory effects of helminth infections may be detected at larval stages which are not reproductively competent, identifying the source of proteins in the *A. suum* secretome could help prioritize proteins for more intensive study: proteins released in uterine fluid, for example, may be hypothesized to be present for maintenance of egg and 1^st^ stage larval viability more than for their effects on the host.

Protein identification in *A. suum* ESP, PE and UF was made more straightforward by the publication of the *A. suum* genome [26]. Comparisons among the compartments illuminate some fundamental aspects of the secretome; in addition, comparison of the current results with data obtained from our work on secretomes of other nematode species that parasitize mammals using the same methods [18] permits a deeper understanding of the biology of proteins secreted by parasitic nematodes.

### Anatomical sources of secreted proteins

Very few tryptic peptides derived from bacteria or of host origin were identified, suggesting that extensive washing eliminated non-nematode proteins from the sample, and that the culture conditions were effectively sterile. The parasites remained fully motile and appeared healthy during the incubation period, suggesting that proteins released from degenerating nematodes were not major contributors to the dataset. Experience in our laboratories suggests that *A. suum* can be maintained without decreases in ATP content or physiological decay for several days under these conditions. Although it is possible that protein release changes upon removal from the host, similar periods of incubation have been used in all other reports of secretome composition; whether the secretome detected in culture is a faithful reproduction of the *in situ* secretome is an area of high priority for research.

Whether any of the nematode proteins detected in ESP were excreted from the intestinal tract cannot be determined based on this analysis. Several types of proteases are abundant in both UF and ESP, but a homolog of a metallo-endoprotease (MEP) protease localized in the intestinal tract of hookworms [34] was found in ESP but not UF, suggesting the possibility that proteins released from the parasite intestine may contribute to the secretome. No cuticle proteins were found in ESP. While release of proteins from the nematode surface has been reported [35], the origin of these proteins is not clear. It seems unlikely that pathways exist for the export of proteins from the hypodermis through the cuticle. Instead, surface-associated proteins may be released from the ES system and adhere to the cuticle after secretion [18]. The absence of classical cuticular proteins in ESP mirrors what has been reported in other nematode secretomes and we conclude that turnover of parasite cuticle is at best a minor source of protein release from healthy nematodes under these conditions.

### Inter-compartmental comparison

Since the secretome contains proteins derived from UF and anatomical ES pathways, we compared the protein composition of PE, UF and ESP. The composition of these three samples was highly overlapping (Figure 1). Significantly, the ESP fraction included a high proportion of proteins also detected in UF. As the majority of UF proteins detected in ESP were among the most abundant in the UF proteome, we assume that all UF proteins would appear in the ESP fraction if a large enough sample was analyzed. It is important to note that non-UF proteins were prominent in the ‘most abundant’ subset of ESP (Table 4), confirming the hypothesis that anatomical secretory pathways provide an important contribution to the secretome. Although 28% of proteins in the ESP-UF subset were also identified in the PE fraction, there was no overlap among the 20 most abundant proteins in these two sets (Table 3–4).

Almost 40% of the proteins detected in UF were present in PE. Based on GO term analysis, the protein functional compositions of PE and UF were much more similar to each other than either was to ESP-UF (Figures 2–5). These results suggest that UF is primarily derived from PE, but that the ESP-UF fraction is derived differently. Proteins unique to ESP also have distinct GO term profiles compared to PE and UF. Unique ESP components include homologs of filarial antigens, some proteases, antioxidant enzymes, hypothetical proteins and some proteins involved in glycogenesis. In contrast, PE unique proteins include other enzymes involved in glycogenesis and antioxidant activities, very few proteases, and proteins involved in binding activity (not shown).

### Cross-species comparisons

To avoid complications arising from the use of different methods, we only included datasets generated in our laboratory. Comparisons were based on the primary annotation of proteins. Based on conservation of proteins, UF from *A. suum* was more closely related to ESP from *B. malayi* than to ESP from *H. polygyrus*. In contrast, ESP from *A. suum* was more closely related to ESP from *H. polygyrus* than to ESP from *B. malayi*. Although the number of species is obviously limited, we suggest that secretome composition may be determined by both phylogeny and predilection site in the host. The figure for *B. malayi* includes ESP from adult females (11%) and males (18%). Interestingly, the protein composition of female *B. malayi* ESP had little similarity to *A. suum* ESP-UF. Male *B. malayi* ESP was as closely related to UF as to ESP from *A. suum* in protein composition. It thus appears that a given protein may be secreted through more than one pathway. The entirety of the secretome, including proteins derived from UF, must be considered when functional roles in the modulation of host responses are investigated.

Although the three species could be differentiated based on protein composition of ESP, they are difficult to distinguish by GO term classification. GO term distribution is dependent on the number of proteins in the secretome, the proportion of hypothetical proteins and the level of annotation. Although the ESP protein composition had somewhat limited similarity among these 3 species based on annotation, they were quite similar in GO terms, suggesting that the general functions associated with proteins in ESP are conserved.

### Exosomes

Exosomes, membrane-bound vesicles produced by eukaryotic cells, are commonly found in extracellular compartments. Considerable evidence points to the involvement of exosomes in cell-cell communication, immune system modulation and tumor progression. Exosomes contain mRNA, microRNA and proteins [36]–[38]. Exosomes have been detected in the secretory system of *C. elegans* [39], but work has not been reported in parasitic species. Exosome-like vesicles produced by the trematodes *Echinostoma caproni* and *Fasciola hepatica* are proposed to modulate host-parasite interactions through their uptake by host cells [40]. Interestingly, the protein composition of *F. hepatica* ESP shares some similarity with *A. suum* ESP and UF, particularly with regard to proteins typically associated with exosomes [41].

Many exosome-associated proteins are present in high abundance in *A. suum* ESP and other parasitic nematodes [see [25]], including proteases, cellular communication proteins, structural proteins, glycolytic enzymes and detoxifying enzymes. In this regard, the protein compositions of *A. suum* PE and UF were very similar, but were distinct from ESP. The number of proteins in PE and UF with signal peptides was similar and low (14% and 19%, respectively), in contrast to those in ESP (40%; Tables S1–S3). Interestingly, PE and UF were relatively enriched in proteins associated with exosomes compared to ESP (Table S5). This could imply that an exosomal secretion is process associated with egg expulsion, or that the protein content of UF is derived, at least in part, by exosome pathways. The lower abundance of exosome-associated proteins in the ESP-UF fraction may indicate that, although exosomes are involved in protein release from anatomical secretory pathways, classical signal peptide-mediate processes are also important. We propose that these data support the previous assertion that protein secretion from parasitic nematodes occurs primarily through exosomal pathways [25]; further work is needed to verify or refute this hypothesis.

### Functional considerations

#### Lipid binding proteins

Parasites must acquire lipids from their host, probably by using lipid-binding proteins [42]–[43]. These proteins are thought to play key roles in small hydrophobic molecule transportation, membrane trafficking, signaling pathways, and regulatory processes [44]. Lipid-binding proteins have been classified as either nematode polyprotein allergens (NPAs) or fatty acid and retinol-binding (FAR) proteins [45]–[48]. The polyprotein ABA-1 allergen, a member of the NPA family [49], was the most abundant protein identified in ESP, PE and UF; its abundance in UF suggests that this may be the primary source of ABA-1 in ESP, although we also detected a close homolog in the secretome of male *B. malayi* (but not microfilariae) [17]. Close homologues are secreted by many other parasitic nematodes (see [25]). In contrast to NPAs, FAR proteins bind small lipids such as vitamin A (among other ligands) and may sequester them [50]. Locally depressed levels of vitamin A may impede renewal of the intestinal mucosa and interfere with functions of neutrophils, natural killer cells and macrophages. Vitamin A deficiency also affects adaptive immunity by hindering antibody production and differentiation of T helper cells and B cells [50].

Vitellogenins (vtgs) are large lipoproteins that function in nematode oogenesis and larval development [51]. The presence of vtgs in the *A. suum* secretome accounts in part for the high percentage of the GO terms “embryo development” and “regulation of growth” in the biological processes category. Although vtgs were more abundant in *A. suum* PE fluid and UF, they were also detected in ESP and in other nematodes, including *H. polygyrus* [19], [20], *M. incognita* [23] and *D. immitis* [25], but perhaps surprisingly not in ESP from female *B. malayi* [17] or *S. ratti* [24]. The source of secreted vtgs is presumed to be UF; whether these proteins act in the host-parasite interface is unknown.

#### Heat shock proteins

Heat shock proteins (HSPs) were among the most abundant proteins in PE and UF and to a lesser extent in ESP and ESP-UF. HSP 70 and other HSPs are commonly found in nematode secretomes [25]. Genes encoding HSPs are highly conserved in parasites, are inducible, and appear to be crucial for parasite survival [52]. Secreted parasite HSPs are immunogenic [53], but the biology in the host of the HSPs detected in the *A. suum* secretome is unknown. It is possible that keeping parasitic nematodes in culture could cause the release of HSPs (and other proteins) due to stress. Alternatively, release of parasite HSPs into the host may modulate host responses to enable and sustain an infection.

#### Other abundant proteins

Although the medium used to culture the parasites was changed twice, major sperm proteins (MSPs) [54] were abundant in ESP and UF. This observation suggests that the worms may have been reproductively competent during the 24 hr incubation. MSPs have commonly been reported from secretomes of other parasitic nematodes; whether this reflects unusual stability of these proteins or their release from storage by adult females fertilized prior to being placed in culture is unknown. It is also possible that sperm were present in UF and not removed by centrifugation; however, we did not detect mitochondrial proteins in the UF fraction, suggesting that disrupted sperm were not a common contaminant to this proteome.

Nematode transthyretin-like proteins show modest homology to transthyretin proteins from vertebrates [55], [56]. Although vertebrate transthyretins are involved in transport of thyroid hormones and vitamin A, transthyretin-like proteins of nematodes are likely to have diverse and different functions [55], [56]. Transthyretin-like proteins were abundant in *A. suum* ESP, PE and UF. Related proteins were reported in ESP of all parasitic nematodes [25]. Multiple genes encoding these proteins are present in *C. elegans* [55], [56], but phenotypes derived from loss-of-function mutations are uninformative. Their roles in initiating and/or maintaining an infection remain obscure.

Hypothetical proteins were present in each compartment at low frequency. Eight hypothetical proteins were shared between ESP and PE and 8 were found in ESP and UF. Two were shared between ESP, PE and UF. The majority of hypothetical proteins had no homologs in other species. Very few matches were found between hypothetical proteins of *A. suum* and *B. malayi* and *H. polygyrus* through BlastP analyses. Much remains to be learned about the basic biology of parasitic nematodes, but we lack convenient platforms to investigate roles that secreted hypothetical proteins play at the host-parasite interface. It will be interesting to discover if the proportion of proteins with no close homologs decreases as genomes are reported from other species of parasitic nematodes.

Parasite proteases are involved in degradation of host proteins during invasion and migration and also take part in establishment and maintenance of long term infections, at least in part through immunomodulation [57], [58]. Aspartyl-, serine-, cysteine-, and metalloproteinases are found in secretions of GI nematodes. The papain superfamily of cysteine proteinases and zinc metalloproteinases is prevalent in nematode secretions [58]. Cysteine and aspartic proteases were previously identified in *A. suum* PE fluid in low abundance, whereas an aminopeptidase was highly secreted [59]. We found metallopeptidases and serine proteases to be abundant in ESP-UF. Aminopeptidase N was the most abundant protease in the secretome, although the neprilysin (M13) family of peptidases and serine carboxypeptidase A were also found in ESP-UF extracts. Because *A. suum* resides primarily in the duodenum, aminopeptidase N might facilitate digestion of proteins which have been partially digested by gastric enzymes [59], enhancing uptake of small peptides by the parasite. A leucine aminopeptidase (LAP; M17 family of metalloendopeptidases; MEP) in ESP was previously identified as secreted by *A. suum*; it was also reported in PE fluid and extracts of various tissues [59]. A homolog of LAP in filarial nematodes, ES-62, is a secreted phosphorylcholine-containing glycoprotein of 62 kDa [60]. ES-62 alters T and B cell proliferation and suppresses cytokines and interferon involved in TH1 immune responses through the phosphorylcholine moiety.

Protease inhibitors are also commonly found in nematode secretomes (see summary table in [25]) and play important roles in immunomodulation among other functions [61]–[63]. Protease inhibitors are well-known from *A. suum* [64], including inhibitors of elastase, chymotrypsin, trypsin, carboxypeptidase A and B, pepsin and gastricsin. Many protease inhibitors were detected in the current study; their roles in internal compartments are unknown, but the biology of protease inhibitors in parasitic nematodes appears to extend beyond possible effects in the host.

*A. suum* uses fermentative metabolism for energy generation. Energy metabolism mainly occurs through pairing phosphorylation to electron transport to drive anaerobic energy generation [65]. This process yields carbohydrate end products [65] and accounts at least in part for the high number related GO terms (Figure 5). Several glycolytic enzymes are associated with exosome-mediated secretion pathways [36], [38] and are typical components of parasitic nematode secretomes [25]. Perhaps surprisingly, glycolytic enzymes were also abundant in UF and to a lesser extent in PE. As noted, the parasites were fully viable during the incubation, so we do not believe that the glycolytic enzymes came from unhealthy worms. Glycolytic enzymes were among the 20 most abundant proteins in UF. The function of secreted parasite glycolytic enzymes demands further experimental investigation, although some are thought to be involved in modulation of host immune responses [66] and many are commonly associated with exosome pathways of protein release.

A recent report describing the protein composition of ESP from larval stages of *A. suum* [66] shows considerable stage-dependent variation. Interestingly, ESP protein composition from intestinal L4 larvae is much more similar to the composition of adult ESP reported here than is the case for other larval stages, suggesting again that there is a strong niche dependence of ESP content, probably reflecting functional roles of ES proteins in modulating local host responses.

### Conclusions

We exploited the unique sensitivity of mass spectrometry combined with the genome of *A. suum* to identify secreted proteins and proteins in the primary internal fluid compartments of the parasite. Using bioinformatic tools to mine GO terms, molecular and biological functions were retrieved and compared between the sources of these proteins. The protein composition profile of ESP differed from those of PE and UF, which were similar to each other. We suggest that proteins in UF are primarily derived from PE, and that a considerable proportion of secreted proteins originate from sources other than the classical secretory system, at least in female parasites. Some proteins in nematode ESP appear to be secreted through multiple pathways.

## Supporting Information

Figure S1 **Distribution of level 2 biological processes GO terms between ESP from** ***A. suum*** **,** ***B. malayi*** **and** ***H. polygyrus*** **.**
(TIF)

Figure S2 **Distribution of level 4 biological processes GO terms between ESP from** ***A. suum*** **,** ***B. malayi*** **and** ***H. polygyrus*** **.**
(TIF)

Figure S3 **Distribution of level 2 molecular functions GO terms between ESP from** ***A. suum*** **,** ***B. malayi*** **and** ***H. polygyrus*** **.**
(TIF)

Figure S4 **Distribution of level 4 molecular functions GO terms between ESP from** ***A. suum*** **,** ***B. malayi*** **and** ***H. polygyrus*** **.**
(TIF)

Table S1 **Proteins identified in** ***A. suum*** **excretory/secretory products (ESP).** “SP”  =  signal peptide. “Y” indicates the presence of signal peptide while “N” indicates a lack of signal peptide. “PIP” and “SC” indicate protein identification probability and sequence coverage, respectively.
(XLSX)

Table S2 **Proteins identified in** ***A. suum*** **perienteric fluid (PE).** “SP”  =  signal peptide. “Y” indicates the presence of signal peptide while “N” indicates a lack of signal peptide. “PIP” and “SC” indicate protein identification probability and sequence coverage, respectively.
(XLSX)

Table S3 **Proteins identified in** ***A. suum*** **uterine fluid (UF).** “SP”  =  signal peptide. “Y” indicates the presence of signal peptide while “N” indicates a lack of signal peptide. “PIP” and “SC” indicate protein identification probability and sequence coverage, respectively.
(XLSX)

Table S4 **Common and unique proteins in** ***A. suum*** **ESP.**
(XLSX)

Table S5 **The presence of common exosome-associated proteins in** ***A. suum*** **ESP.** Exosome proteins were obtained from [http://www.exocarta.org/exosome_markers]. Comparisons were made using the protein name. Matches were confirmed by sequence alignment.
(XLSX)
