# Supplementary figures and images for: Proteomic Analysis of Adult Ascaris suum Fluid Compartments and Secretory Products
Source: PLoS Negl Trop Dis. 2014 Jun 5;8(6):e2939. doi: 10.1371/journal.pntd.0002939 (PMC4046973; doi:10.1371/journal.pntd.0002939)

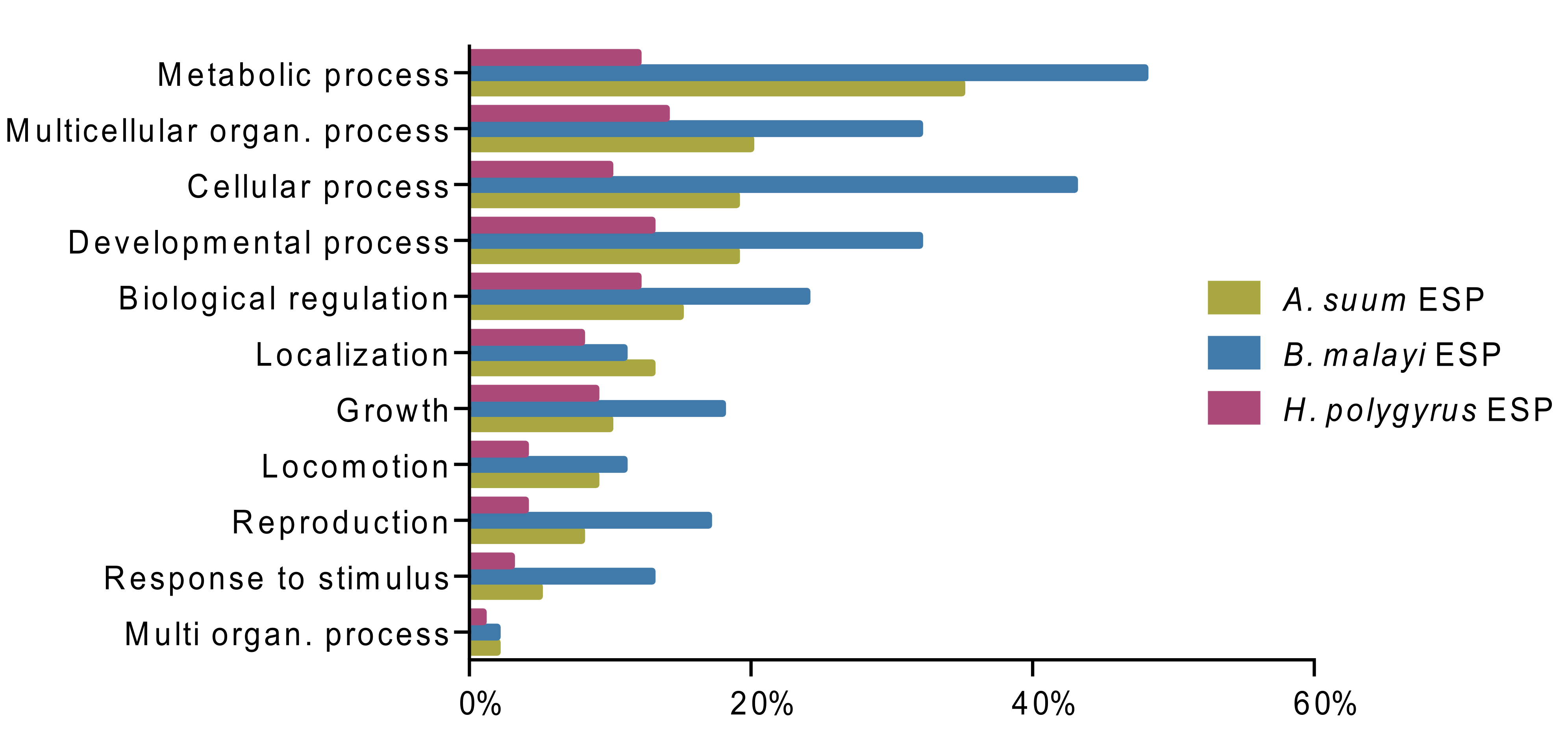

Supplement: Figure S1 — Distribution of level 2 biological processes GO terms between ESP from A. suum , B. malayi and H. polygyrus . (TIF) [file pntd.0002939.s001.tif]

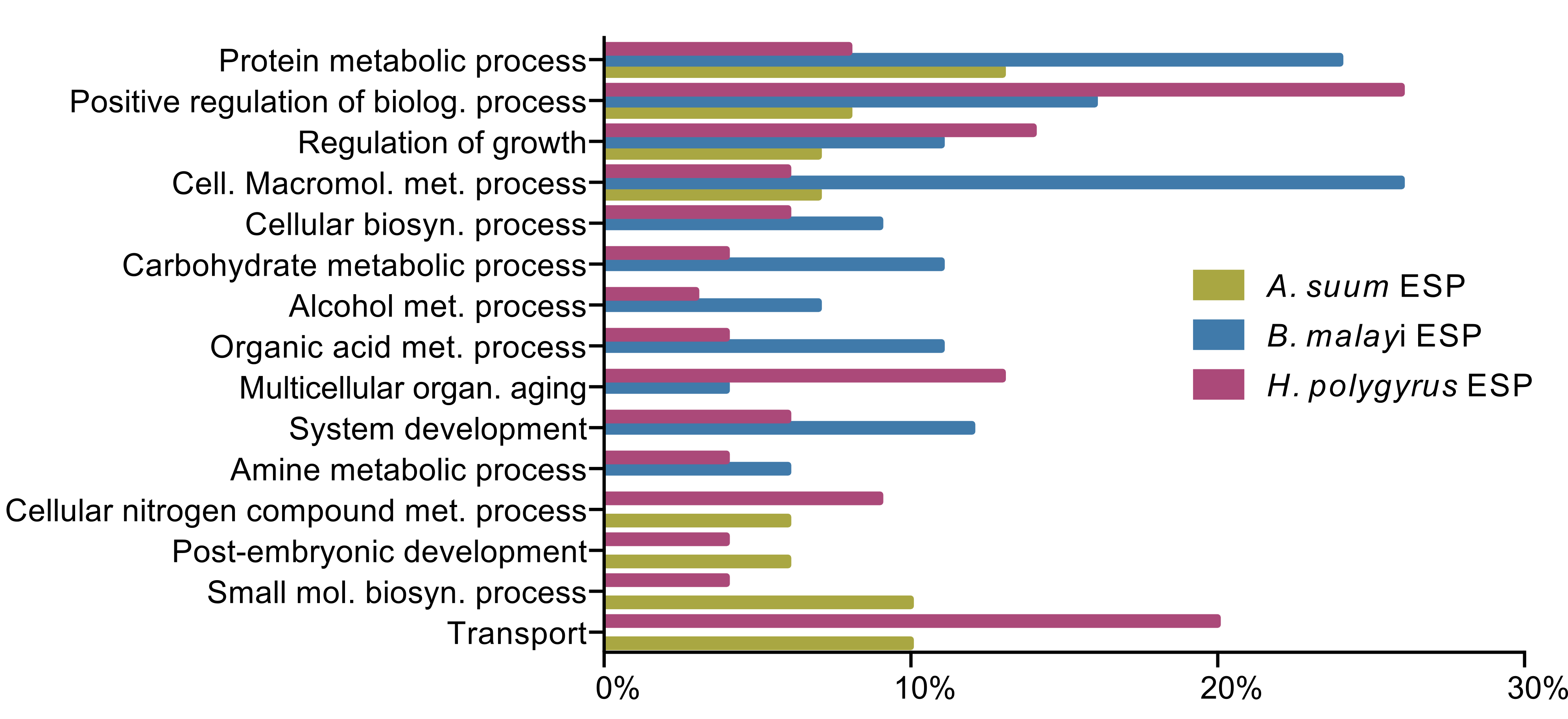

Supplement: Figure S2 — Distribution of level 4 biological processes GO terms between ESP from A. suum , B. malayi and H. polygyrus . (TIF) [file pntd.0002939.s002.tif]

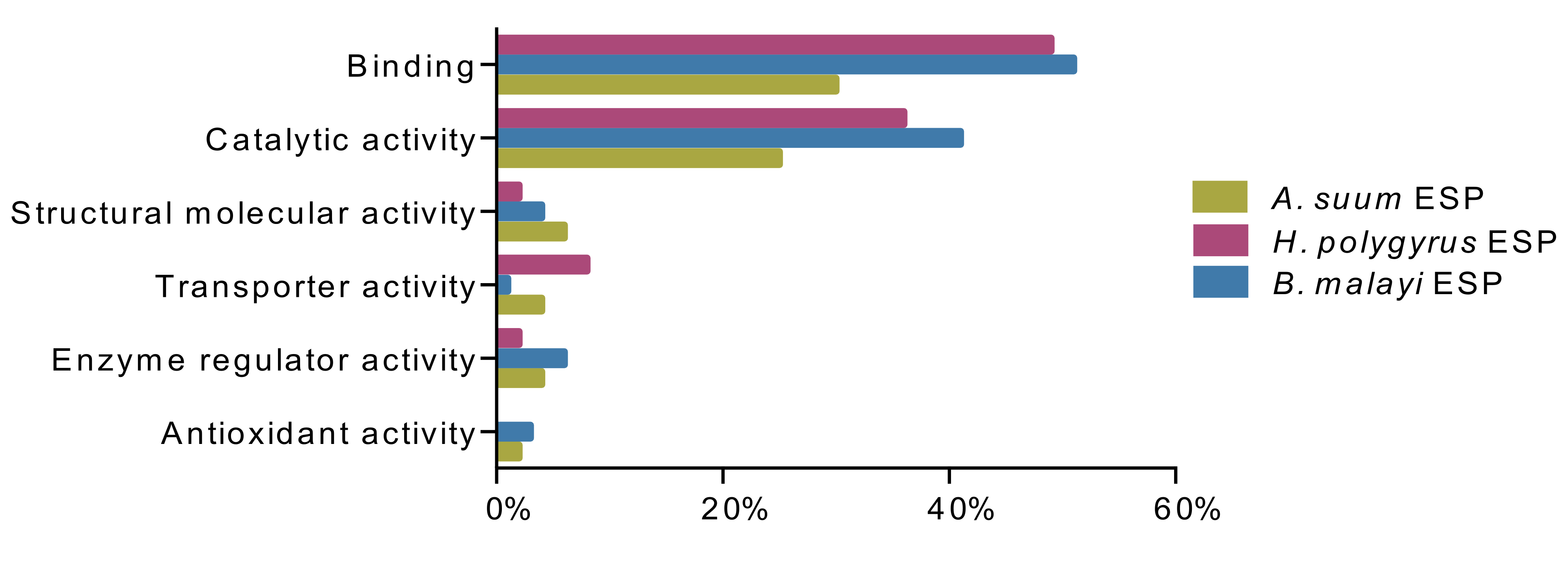

Supplement: Figure S3 — Distribution of level 2 molecular functions GO terms between ESP from A. suum , B. malayi and H. polygyrus . (TIF) [file pntd.0002939.s003.tif]

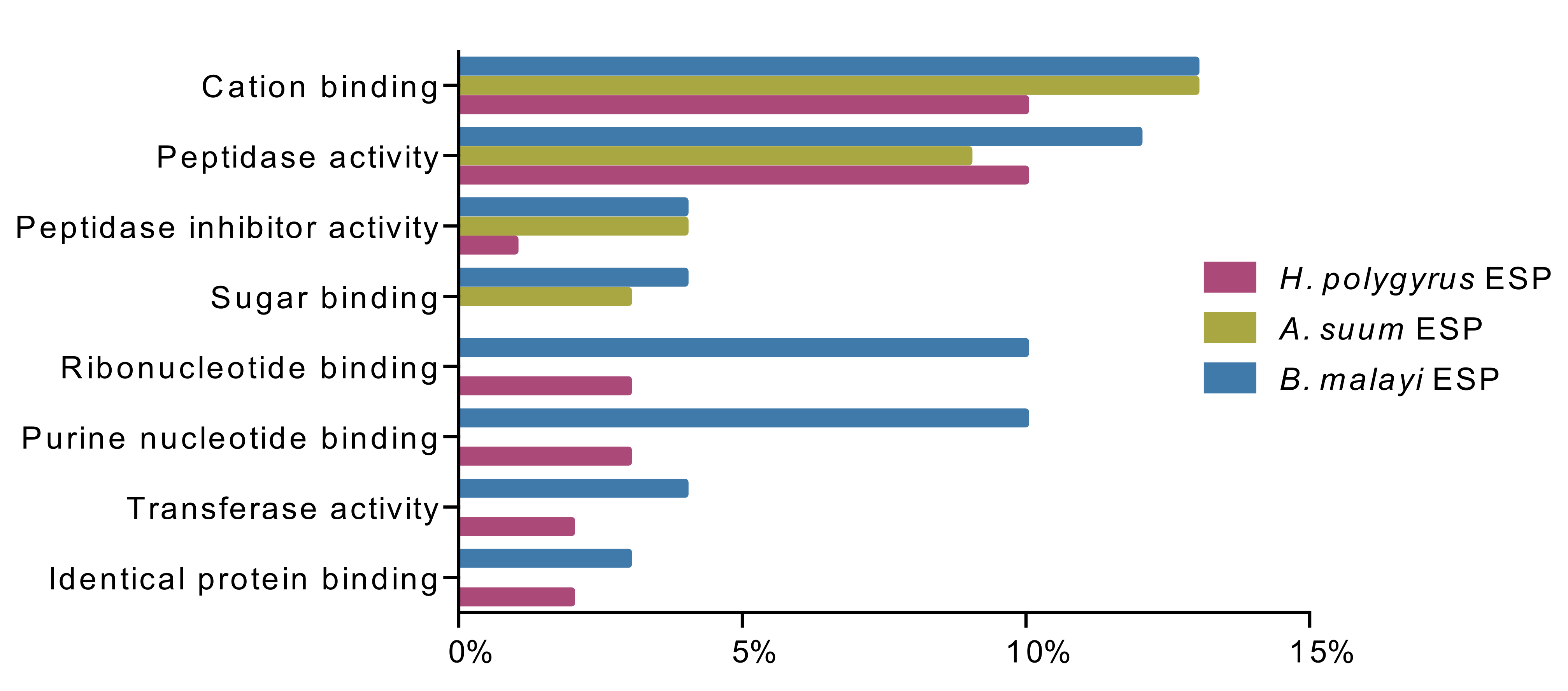

Supplement: Figure S4 — Distribution of level 4 molecular functions GO terms between ESP from A. suum , B. malayi and H. polygyrus . (TIF) [file pntd.0002939.s004.tif]
